# The Tumor-Specific Expression of L1 Retrotransposons Independently Correlates with Time to Relapse in Hormone-Negative Breast Cancer Patients

**DOI:** 10.3390/cells11121944

**Published:** 2022-06-16

**Authors:** Enrico Berrino, Umberto Miglio, Sara Erika Bellomo, Carla Debernardi, Alberto Bragoni, Annalisa Petrelli, Eliano Cascardi, Silvia Giordano, Filippo Montemurro, Caterina Marchiò, Tiziana Venesio, Anna Sapino

**Affiliations:** 1 Candiolo Cancer Institute, FPO-IRCCS, 10060 Candiolo, Italy; enrico.berrino@ircc.it (E.B.); umberto.miglio@ircc.it (U.M.); saraerika.bellomo@unito.it (S.E.B.); alberto.bragoni@ircc.it (A.B.); annalisa.petrelli@unito.it (A.P.); eliano.cascardi@ircc.it (E.C.); silvia.giordano@unito.it (S.G.); filippo.montemurro@ircc.it (F.M.); caterina.marchio@unito.it (C.M.); anna.sapino@unito.it (A.S.); 2 Department of Medical Sciences, University of Turin, 10124 Turin, Italy; carla.debernardi@unito.it; 3 Department of Oncology, University of Turin, 10124 Turin, Italy

**Keywords:** L1 retrotransposons, tumor-exclusive biomarker, breast cancer, basal-like

## Abstract

Background: Long-Interspersed Nuclear Element (L1) retrotransposons are silenced in healthy tissues but unrepressed in cancer. Even if L1 reactivation has been associated with reduced overall survival in breast cancer (BC) patients, a comprehensive correlation with clinicopathological features is still missing. Methods: Using quantitative, reverse-transcription PCR, we assessed L1 mRNA expression in 12 BC cells, 210 BC patients and in 47 normal mammary tissues. L1 expression was then correlated with molecular and clinicopathological data. Results: We identified a tumor-exclusive expression of L1s, absent in normal mammary cells and tissues. A positive correlation between L1 expression and tumor dedifferentiation, lymph-node involvement and increased immune infiltration was detected. Molecular subtyping highlighted an enrichment of L1s in basal-like cells and cancers. By exploring disease-free survival, we identified L1 overexpression as an independent biomarker for patients with a high risk of recurrence in hormone-receptor-negative BCs. Conclusions: Overall, L1 reactivation identified BCs with aggressive features and patients with a worse clinical fate.

## 1. Introduction

The “Junk DNA” definition was coined to describe the large fraction of the non-coding, redundant and repeated human genome [1,2]. The Long-Interspersed Nuclear Element (LINE-1 or L1) sequences are randomly widespread and belong to this portion of human DNA, representing about 18% of the total amount. These repeated sequences (about 500,000 repetitions) belong to the family of transposable elements, with a unique self-induced “copy and paste” mechanism through the transcription and translation of two open reading frames (ORFs) (ORF1 and ORF2) [3,4,5]. In particular, ORF2 exhibits a retro-transcriptase activity, thus leading to the label of “retrotransposons”. During their life cycle, L1s are transcribed and translated through the standard cellular apparatus, and the two ORF-encoding proteins lead to the production of a cDNA molecule that is subsequently reintegrated into a genomic site different from the original one. Nevertheless, this cycle rarely closes because of the presence of frequent truncating mutations scattered along most of the L1s or the involvement of different L1-silencing mechanisms, among which promoter methylation is the best known [6].

In healthy adult human cells, this process is negatively monitored, and retro-transpositions are rarely identified. However, in stressing cell conditions such as cancer, the deregulation of global DNA methylation favors L1 reactivation [7,8]. Alongside the “historical” carcinogenic insertions of L1 reported in genes such as *APC* (in a colon cancer patient) [9] and *MYC* (in a breast tumor patient) [10], L1 sequences have been widely exploited as a surrogate prognostic biomarker of global methylation in different cancer types [11]. L1 reactivation can be monitored by evaluating mRNA/protein expression of the ORFs [12] or the L1 de novo insertions [13]. Confirming previous evidence [11], L1 de novo insertion was reported in colon, lung and breast cancers (BC) [14]. Accordingly, preclinical studies have also described an increase in L1 expression in BC cells [15,16] and tumors [17], with potential clinical implications. In this regard, the pioneering work by Chen and colleagues clearly showed a correlation between L1 protein expression and aggressive features in a cohort of 95 BCs, together with a lack of expression in tumor-free mammary glands [17]. Despite this, many questions about the clinicopathological characteristics of BC patients with L1 sequence reactivation are still open.

To assess the poorly known correlation between L1 reactivation and the clinical and molecular characteristics of breast carcinomas, we evaluated the expression of these retrotransposons in pre-clinical and clinical settings, investigating BC and non-transformed mammary epithelial cells along with a cohort of 210 BCs and 47 normal mammary glands. To this purpose, we assessed the expression of L1 transcript (mRNA) by using two independent qRT-PCR assays encompassing the ORF1 and ORF2 sequences. The mRNA expression results were then compared with several molecular and clinical data, including promoter methylation, molecular subtyping and disease-free survival (DFS).

## 2. Materials and Methods

### 2.1. Cell Lines

We profiled 14 cell lines, 2 non-transformed and 12 breast cancer cells. HMEC non-transformed cells were grown in DMEM/F15 with 10% FCS plus 10 ng/mL insulin, 0.5 μg/mL hydrocortisone and 5 ng/mL EGF. MCF-10A cells were grown in DMEM/F12 with 5% HS, 10 μg/mL insulin, 10 ng/mL EGF and 500 ng/mL hydrocortisone. MDA-MB-453 cells were grown in Leibovitz’s L-15 Medium (ThermoFisher Scientific, Waltham, MA, USA) with 10% FBS (Biowest, Nuaillé, France) in free-gas-exchange atmospheric air; SKBR3 and MDA-MB 231 in high-glucose DMEM (Sigma-Aldrich, Saint Luis, MO, USA) with 10% FBS; MCF-7 in high-glucose DMEM with 10% FBS supplemented with 10 µg/mL insulin; T47D, ZR751, MDA-MB 468, BT549, HS-578T, HCC70 and HCC1187 in RPMI (Sigma-Aldrich) with 10% FBS; BT474 in RPMI with 20% FBS and 10 µg/mL insulin. MDA-MB-468, T-47D, HS-578T, MDA-MB-231 and BT549 cell lines were obtained from the NCI-60 panel, while MDA-MB-453, SKBR3 and ZR751 cells were derived from the Interlab Cell Line Collection (ICLC) and MCF-7, HCC70, HCC1187, HMEC and MCF-10A from the American Type Culture Collection (ATCC). Lastly, BT474 cells were obtained from the “Deutsche Sammlung von Mikroorganismen und Zellkulturen” (DMSZ, Braunschweig, Germany). BC cell lines are reported in Supplementary Table S1.

The genetic identity was confirmed by short tandem repeat profiling (PowerPlex^®^ 16 HS System, Promega, Madison, WI, USA) evaluated in September 2021.

### 2.2. Breast Cancer Patient Cohort

We analyzed a total of 210 retrospective BCs collected at the Candiolo Cancer Institute. All patients signed a written informed consent (Ethical committee of FPO-IRCCS Candiolo Cancer Institute, protocol code: PROFILING #001-IRCC-00IIs-10, approved on the 2 November 2011), as reported [18]. Patient clinical and pathological features are reported in Supplementary Table S2. Data about hormone receptor (HR) status (expression of estrogen and progesterone receptors, ER and PgR, respectively), HER2 expression and tumor proliferation levels (Ki67 labelling index), as well as details related to histologic type, stage (pT and pN status) and tumor grade were retrieved from pathology reports. Tumor infiltrating lymphocytes (TILs) were assessed following the International Recommendations of TILs scoring [19]. Information about relapse was obtained for all the patients, allowing the assessment of disease-free survival (DFS). As controls for L1 expression, we analyzed 47 normal mammary gland tissues, derived from prophylactic mastectomy.

### 2.3. Nucleic Acids Extraction

DNA was extracted from cells using the Blood & Cell Cultured Mini Kit (Qiagen, Hilden, Germany), while the RNeasy Mini Kit (Qiagen, Hilden, Germany) was used for RNA extraction. TURBO^™^ DNase (ThermoFisher Scientific, Waltham, MA, USA) treatment was additionally performed, according to the manufacturer’s protocol, to avoid the problem of genomic contamination in qRT-PCR.

After review with hematoxylin and eosin (H&E), FFPE tumoral sections were dissected to obtain nucleic acids from tissue areas containing at least 100 cells with >50% tumoral cells. Maxwell RSC FFPE RNA Kit (Promega) was used to obtain RNA from the 210 FFPE BC samples and the 47 FFPE BC normal mammary glands. Genomic DNA was purified from a subset of 41/210 FFPE tissues using the GeneRead DNA FFPE Kit (Qiagen, Hilden, Germany). Nucleic acids were quantified with the Qubit^®^ 2.0 fluorometer (ThermoFisher Scientific, Waltham, MA, USA).

### 2.4. qRT-PCR of L1-ORF1 and ORF2 Expression

One μg of purified RNA was reverse-transcribed in a mix containing both oligo(dT) and random hexamers using SuperScript^™^ IV VILO^™^ reverse transcriptase (ThermoFisher Scientific, Waltham, MA, USA).

The expression of L1-ORF1 and ORF2 transcripts were evaluated in triplicate by qRT-PCR using two independent couples of primers and specific thermal profiles, reported in Supplementary Table S3. Primers were designed using the L1 reference sequence (GenBank accession number: AH005269.2). Relative expression quantification (RQ) was calculated using GAPDH and 18S as endogenous controls (ECs) and the following formula: RQ = 2[ΔCt], where ΔCt = [Cttarget gene—CtECs]. For FFPE tumor tissues, we used the expression of L1-ORF1 and ORF2 in normal breast glands (NBs) as a normalizer, so we applied the RQ to the ΔΔCt, with the formula RQ = 2 − [ΔΔCt], where ΔΔCt = BC [Cttarget gene—CtECs] − NBs [Cttarget gene—CtECs].

### 2.5. PAM50 Subtyping

Molecular subtyping was performed using the Prosigna^®^ assay (NanoString Technologies Inc., Seattle, WA, USA) based on the classification algorithm PAM50 [20,21,22,23,24] and the analysis system Dx nCounter on BC cells and on the 210 BC FFPE tissues. Briefly, 250 ng was used for the assay. For each sample, 10 µL of the Hybridization Buffer, 5 µL of the Reporter CodeSet and 5 µL of the ProbeSet were used in the hybridization reaction, which took place at 65 °C overnight. RCC data were shared to NanoString to apply the PAM50 private algorithm and assign the specific BC molecular subtype.

### 2.6. Mutational Analyses

The 41 BCs with available DNA were mutationally characterized with a 147-hotspot breast cancer gene panel using the MassARRAY^®^ system (Agena Bioscience, San Diego, CA, USA) based on the MALDI-TOF (matrix-assisted laser desorption ionization time-of-flight) method, as previously reported [25].

### 2.7. L1 Promoter Methylation Analysis

L1 methylation was studied for both cancer cells and the 41 BCs with available DNA by performing quantitative bisulfite pyrosequencing in triplicate using the PyroMarkQ96 pyrosequencing (Qiagen, Hilden, Germany), as previously reported [26]. Briefly, for each sample, 300 ng of DNA was converted by MethylEdge Bisulfite Conversion System (Promega, Madison, WI, USA). Five μL of each converted DNA sample was amplified with the thermal profile, primers (designed using the L1 reference sequence GenBank accession number: X58075) and amplification mix reported in Supplementary Table S3. The primed single-stranded DNA templates were subjected to pyrosequencing by using the following primer: L1 seq 5′-GGTGTGGGATATAGTT-3′, using the PyroMarkQ96 software (v.2.5), which analyzed the following sequence: TT/CGTGGTGT/CGTT/ CTTTT/CTTAAGTT/CGGTTTGAAAAGT/C through the following dispensation order: A TCAGTGTGTCAGTCAGTCTCAGTCAGTGAGTC. Methylation levels were obtained considering C/T in positions 2, 3, 5 and 6 of the pyrograms. An overall L1 methylation level was calculated as the average of the proportion of C (%) at the 4 CpG sites. The fourth position was adopted as a site of control.

### 2.8. Statistical Analysis

Data analysis was carried out with the SPSS version 20.0 software (IBM, Armonk, NY, USA) by three independent authors (E.B., C.D., S.E.B.).

Student’s t-distribution was used to determine the distribution of L1-ORF1 and L1-ORF2 expression and the Fisher’s Exact Test to evaluate contingency tables for nominal variables. Survival analysis of disease-free survival (DFS) was evaluated with the Kaplan–Meier method, and groups were compared with the log-rank test. DFS was calculated as the interval between the beginning of the therapy to the time of progression. The Cox regression model (logistic regression, backward) was used to perform the multivariate analysis for tumor relapse. All the available *p*-values are reported within the text and/or in the tables. *p* < 0.05 was considered statistically significant. ROC curves were produced with the pROC package (R v4.0.2).

## 3. Results

### 3.1. L1 Expression in Breast Cancer Cell Lines

**Figure 1:**
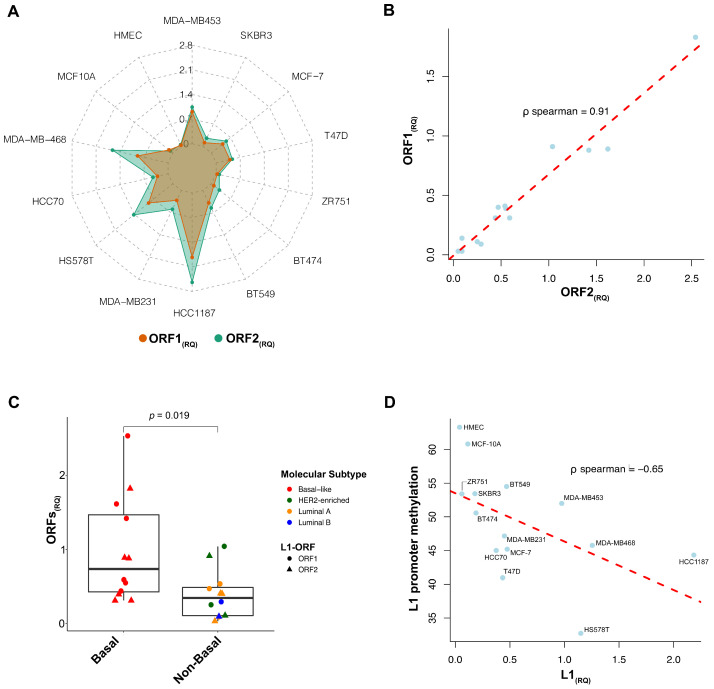
Representation of LINE-1 (L1) expression in Breast Cancer (BC) cell lines. (**A**) Spider plot with double-variable Y-axis, reporting both Open Reading Frame relative quantification ORF1(RQ) (coral) and ORF2(RQ) (sea green) values. A higher level of L1 mRNA was detected in cancer cells, compared to the non-transformed, with heterogeneity in ORFs amount. (**B**) Correlation plot reporting the association between ORF1 and ORF2 within the cells (blue dots). Red dashed trending line underlines the positive correlation. (**C**) Boxplot of the L1 expression in BC cells, clustered for the molecular subtype. Basal cells showed a significantly higher level of both ORF1 (circles) and ORF2 (triangles). Non-basal showed heterogeneity, with a single HER2-enriched cell line (MDA-MB453) showing higher levels of both ORFs. (**D**) Correlation plot reporting the association between L1 expression (as the mean level between ORF1 and ORF2, for each cell) and the L1-promoter methylation (blue dots). Red dashed trending line underlines the negative correlation.

We first assessed L1 expression by evaluating the relative quantification RQ (ΔCt) of ORF1 and ORF2 in twelve BC and two non-transformed mammary gland cell lines. L1 transcript levels were barely detectable in the non-transformed mammary gland cells, namely, HMEC and MCF-10A (ORF1_(RQ)_: 0.05 and ORF2_(RQ)_:0.03; ORF1_(RQ)_: 0.08 and ORF2_(RQ)_: 0.09, respectively), whereas all BC cells showed higher but heterogeneous levels of both ORF1 and ORF2 (ORF1_(RQ)_mean: 0.59, range 0.03–1.83; ORF2_(RQ)_mean: 0.81, range 0.09–2.54, Figure 1A). ORF1 and ORF2 expression levels were highly correlated (*p* < 0.01, ρ Spearman = 0.91, Figure 1B). Basal-like cells displayed the highest RQ values; in particular, the HCC1187, MDA-MB468 and HS-578T cells had the highest content of L1-ORFs. Conversely, Luminal cells homogeneously showed small amounts of L1 transcripts. Of note, the two HER2-enriched BC cell lines showed opposite levels of L1-ORF expression (MDA-MB453 were L1-expressive, whereas SKBR3 were L1 non-expressive cells). By dichotomizing cells as basal-like versus non-basal, L1-ORF levels were significantly higher in the basal-like group (*p* = 0.019, Figure 1C). Interestingly, we identified a trend of L1 enrichment in BC cells with the highest metastatic potential (defined from [27,28,29]) (*p* = 0.09, Supplementary Table S1).

When analyzing L1-promoter methylation, the non-transformed cells displayed methylation levels higher than 60% (HMEC = 61%, MCF-10A = 63%), which is the cut-off to discriminate methylation/hypomethylation [30,31], whereas all the BC cells exhibited variable levels of promoter demethylation (mean: 47%, range: 33–55%). We observed an inverse correlation between L1-ORF expression and promoter demethylation (*p* = 0.01, ρ Spearman = −0.65) (Figure 1D).

### 3.2. L1 Expression in Breast Cancer Tissues

**Figure 2:**
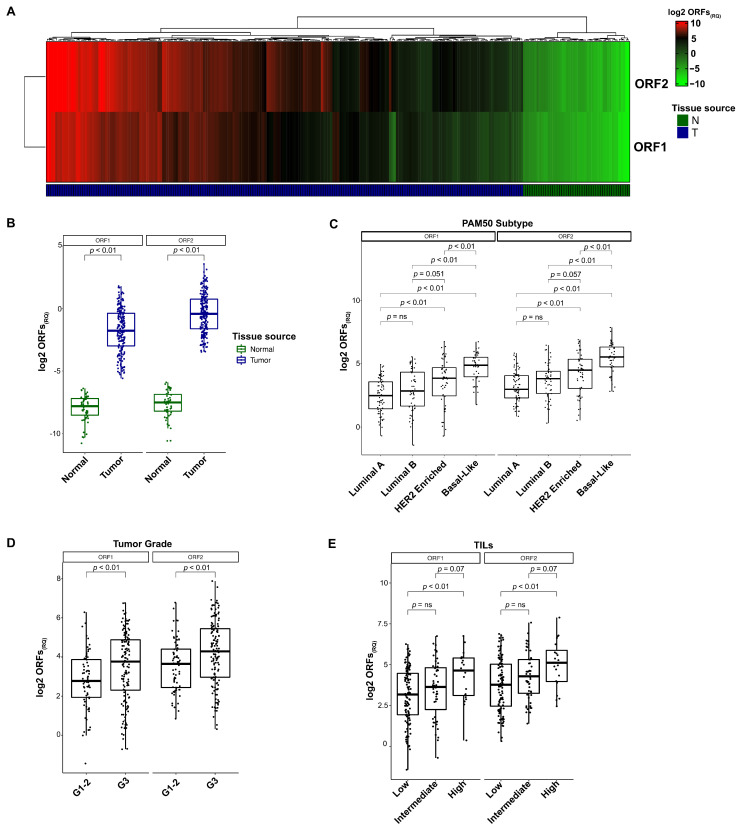
Representation of L1 expression in BC patients (*n* = 210). (**A**) Heatmap of ORF1_(RQ)_ and ORF2_(RQ)_ within both breast normal glands and tumor. Green-to-red scale for the log2 expression revealed a substantial absence of L1 expression in the normal tissues (dark green annotated), compared to the highly heterogeneous level of expression in breast lesions (dark blue annotated). (**B**) Paired boxplot of the L1 expression (log2 ORFs_(RQ)_) in BCs compared to the breast normal tissues. Color-coded boxes revealed a significantly higher expression of L1 in tumors. (**C**) Paired boxplot of the L1 expression (log2 ORFs_(RQ)_) across the BC subtypes, with an enrichment of ORFs amount in basal-like tumors and strong heterogeneity in HER2-enriched lesions. (**D**) Paired boxplot of the L1 expression (log2 ORFs_(RQ)_) stratified by tumor grade. L1 expression was higher in dedifferentiated tumors. (**E**) Paired boxplot of the L1 expression (log2 ORFs_(RQ)_) across the BCs, divided for lymphocyte content, low (<10% of TILs), intermediate (10% < x < 30% of TILs) and high (>30% of TILs).

To confirm the BC-exclusive expression of ORF1 and ORF2, we compared the RQs (ΔCt) of both transcripts in 210 BCs and 47 normal mammary glands. Unsupervised clustering revealed a clear separation between BCs and non-tumoral tissues, the latter being characterized by barely detectable levels of expression for both ORFs (ORF1_(RQ)_: 0.05, range: 0.005–0.09; ORF2_(RQ)_: 0.06, range: 0.006–0.07, Figure 2A).

None of the tumor samples showed an ORF amount lower than the most expressive normal tissue (ORF1_(RQ)_: 0.55, range: 0.11–3.47; ORF2_(RQ)_: 1.32, range: 0.09–11.75), supporting the tumor-specific expression of both transcripts (ORF1 and ORF2 both *p* < 0.01, Figure 2B). In line with BC cells, we detected a significant heterogeneity of L1 mRNA expression within the BC cohort (Figure 2A). To decipher this complexity, we assessed whether L1 expression was enriched in tumors with specific clinical and pathological features. To do this, the RQ values were calculated by normalizing the level of ORF1 and ORF2 obtained in BC samples with the baseline amount of the mRNA detected in normal lesions (ORF1_(RQ)_ mean: 17.79, range: 0.37–108.13; ORF2_(RQ)_ mean: 27.69, range: 1.24–235.24).

Subsequently, the cohort of 210 BCs was stratified according to PAM50 intrinsic molecular subtype and histopathological features. Clinicopathological data and statistics are reported in Table 1.

Each of the PAM50 molecular subtypes had a comparable number of patients (67/210 were Luminal A, 32%; 51/210 were Luminal B, 24%; 52/210 were HER2-enriched, 25%; 40 were basal-like, 19%). Similar to the scenario detected in the BC cell lines, we observed the enrichment of L1 transcripts in the basal-like subtype (ORF1_(RQ)_mean: 34.54, range: 3.43–107.49; ORF2_(RQ)_mean: 58.76, range: 7.10–235.24) (Figure 2C). Luminal BCs (both A and B) showed a reduced expression of L1s, with 82% of Luminal tumors presenting ORF1 and ORF2 values below the mean level of expression (97/118) (Figure 2C). The HER2-enriched subgroup showed the highest variability, with 55% of tumors characterized by L1 expression levels close to the Luminal group (29/52) and 45% with levels superimposable to basal-like BCs (23/52). The mean L1-ORF_(RQ)_ for each subgroup is reported in Table 1.

L1-ORF expression was increased in poorly differentiated (G3) BCs compared to G1/2 tumors (ORF1 and ORF2 both *p* < 0.01) (Figure 2D). BCs characterized by a high Ki67 level showed an increased expression of both ORFs compared with those tumors with Ki67 < 20% (ORF1_(RQ)_ *p* < 0.01 and ORF2_(RQ)_ *p* < 0.01) (Table 1). Following the stratification of BCs into three subsets according to their tumor-infiltrating lymphocytes (TILs) level (low: TILs < 10%, intermediate: 10% < TILs < 30% and high: TILs > 30%), we found significant variability in the L1 mRNA distribution among the subgroups (ORF1_(RQ)_ *p* = 0.02, ORF2_(RQ)_ *p* = 0.01), with a higher L1 expression in tumors with higher TIL scores (Figure 2E). The mean L1-ORFs_(RQ)_ for each group is reported in Table 1.

Finally, to confirm the correlation between L1 demethylation and the expression observed in BC cells, we assessed L1-promoter methylation in a subset of 41 BCs, balanced for the PAM50 molecular subtypes (Luminal A: *n* = 8, Luminal B: *n* = 11, basal-like: *n* = 12, and HER2-enriched: *n* = 10). In addition to a mean methylation percentage of 57% (range 27–82%), we observed a negative correlation between demethylation and ORF2 expression (*p* = 0.01, Spearman correlation coefficient: −0.56), together with a weaker trend for ORF1 (*p* = 0.06, Spearman correlation coefficient: −0.3) (Supplementary Figure S1A,B). Hotspot mutational profiling of this subset identified twelve *TP53* (29%), eight *PIK3CA* (20%), two *AKT1* (5%), and one CDH1 pathogenic variants (2%) (Supplementary Table S4). Seventeen patients did not carry any alteration (WT) in the nucleotide positions included in the panel. Interestingly, TP53 mutated patients showed higher levels of L1 expression, compared with both WT and *PIK3CA*/*AKT1* mutated tumors (ORF1 *p* = 0.01, ORF2 *p* < 0.01) (Supplementary Figure S1C).

### 3.3. L1 mRNA Enrichment Was Associated with Relapse and Shorter Disease-Free Survival

We collected tumor relapse data for 208/210 BC patients, including the time from the primary treatment to the recurrence, defined as the disease-free survival (DFS). A heatmap for both ORF1_(RQ)_ and ORF2_(RQ)_ showed a trending polarization of the patients with recurrence according to high L1 expression (Supplementary Figure S2A). Patients with tumor relapse showed higher expression of both L1-ORFs (*p* < 0.01, Table 1). Similarly, ROC curves analysis for both ORFs defined a good level of accuracy in identifying patients with relapse (ORF1 AUC = 0.79, Youden index = 11.3; ORF2 AUC = 0.82, Youden index = 15.2) (Supplementary Figure S2B).

**Figure 3:**
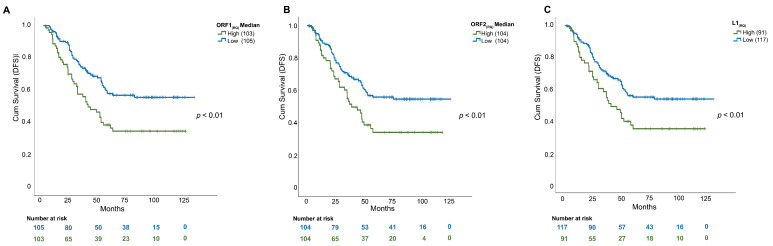
Disease-free survival curves for 208 BC patients, stratified by L1 expression. (**A**) DFS curve for ORF1 low expressor (<ORF1_(RQ)_ median value), ORF1 high expressor (>ORF1_(RQ)_median value). High expressors show a shorter DFS compared with low expressors. (**B**) DFS curve for ORF1 low expressor (<ORF1_(RQ)_ median value), ORF2 high expressor (>ORF1_(RQ)_median value). High expressors show a shorter DFS compared with low expressors. (**C**) DFS curve for L1 low expressor (ORF1_(RQ)_ or ORF2_(RQ)_ below the median value), L1 high expressor (both ORF1_(RQ)_ or ORF2_(RQ)_ above median value). High expressors show a shorter DFS compared with low expressors.

We then divided the cohort into ORF1/ORF2 “high”- and “low”-expressors (considering both median and average RQs as cut-offs) to assess whether L1 expression was associated with differential DFS. As shown in Figure 3A, a significantly shorter DFS was detected considering the ORF1_(RQ)_ median level as the cut-off. The BCs highly expressing ORF1 were characterized by a median of 32 months of DFS compared to 49 months for the low expressors (*p* < 0.01), with a two-times higher risk (hazard ratio, HR) of relapse. Similarly, high ORF2 values identified patients with an increased risk of relapse (*p* < 0.01, HR: 1.9, median low: 48 months vs. median high: 36 months, Figure 3B).

Data concerning the stratification with the mean levels of ORF1 and ORF2 expression are reported in Table 2.

Subsequently, we questioned whether merging the L1-ORF1 and ORF2 results would allow us to simplify the stratification of patients with relapse. The group of “L1 high-expressors” comprised patients with both ORF1/ORF2_(RQ)_ values over the median level, whereas “L1 low-expressors” included patients with at least one ORF_(RQ)_ below the median level. The derived Kaplan–Meier curve identified a strong level of significance (*p* < 0.01), with an HR for L1 high expressors of 1.9 (median survival high: 36 months, low: 48 months) for both cut-offs (Figure 3C and Table 2).

To assess whether L1 can be an independent prognostic factor, we applied the Cox regression model for multivariate analysis. Covariates in this analysis included L1 expression, tumor grade (G1–2 vs. G3), node involvement (0 vs. 1–3 nodes vs. >3 nodes), Ki67 expression (high vs. low), PAM50 subtypes, TILs (low, medium and high) and tumor size. Of note, only L1 expression (both stratified for average and median) and lymph-node involvement retained an independent level of significance (Table 2).

**Figure 4:**
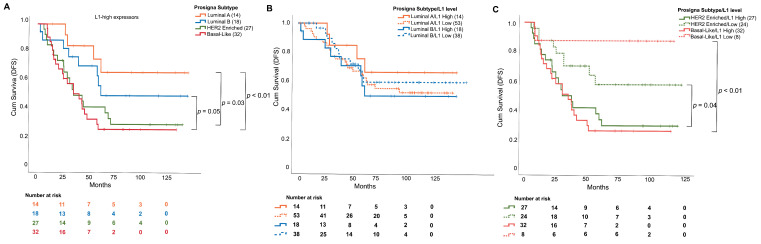
Disease-free survival curves of the BCs stratified for L1 expression and for the PAM50 molecular subtype. (**A**) DFS curve only for L1 high expressor (both ORF1(RQ) or ORF2(RQ) above median value), stratified for the molecular subtype. Basal-like and HER2-enriched tumors showed a reduced DFS. (**B**) DFS curve for Luminal tumors (Luminal A: orange, Luminal B: blue), divided for L1 high (solid lines) or low (dashed lines) expression (median cut-off). No differences were identified for the different L1-expressive groups. (**C**) DFS curve for basal-like (red) and HER2-enriched (blue) tumors, divided for L1 high (solid lines) or low (dashed lines) expression (median cut-off). High expressors show a shorter DFS compared with low expressors.

Since we described increased L1 mRNA expression as a negative prognostic factor, we focused on the DFS of L1 high expressors (cut-off: median L1(RQ)). In this subgroup, patients with a basal-like (median: 46 months) or with a HER2-enriched subtype (median: 52 months) had a worse prognosis, whereas Luminal patients with a tumor showing high L1 expressions were characterized by a longer DFS (Luminal A: 92 months, Luminal B: 75 months, *p*-values, HR and CI95% in Table 3, Figure 4A). Both Luminal A and Luminal B BCs displayed no differences between L1 high and L1 low expressors in terms of DFS (Figure 4B). Conversely, basal-like or HER2-enriched patients showed a clear DFS stratification considering L1 expression, in which lower ORF expressors displayed a better outcome (Figure 4C).

Finally, by evaluating the correlation between L1 expression and the type of therapy, we identified a significantly higher L1 amount in patients treated with chemotherapy who experienced tumor relapse (29/50), compared with those without recurrence (21/50) (*p* = 0.03 for both ORFs). No other correlations were identified between L1 expression and recurrence in other therapeutic approaches (hormonal therapy + chemotherapy, hormonal therapy alone, anti-HER2 drugs, Supplementary Table S5).

## 4. Discussion

LINE-1 (L1) sequences are usually silenced in human tissues through several mechanisms, including hypermethylation of their promoter [32]. Deregulation of these mobile elements has been widely investigated [33,34,35] in several neoplasms [10], in particular, their promoter methylation level as a surrogate for global methylation [30,31], the L1 de novo insertion for gene disruption [14] and L1 expression.

Here, we report for the first time a detailed characterization of RNA-based L1-ORF expression in a large cohort of BC patients in the attempt to contextualize L1 transcription among BC patients and to correlate L1 reactivation with clinical outcome. We demonstrated a high level of tumor-specific L1 transcripts in 12 BC cell lines and in a retrospective BC cohort, enhanced in HR-negative BCs. Moreover, the association between ORF expression and a high risk of tumor recurrence, confirmed by multivariate analysis, proposes L1 as a biomarker for BC prognostic stratification.

The tumor-specific activation of L1 mRNA can be considered crucial to define L1 as a biomarker [36]. The absence of L1 expression in the two breast normal cell lines and in the 47 mammary glands analyzed was in line with previously reported higher methylation levels in normal breast samples [37] and the absence of ORF1p/ORF2p IHC staining in both normal and peritumoral tissues [17]. We pursued the assessment of L1 mRNA instead of L1 methylation and protein expression for both technical and biological reasons. The identification of an L1 expression threshold value to distinguish tumor from normal tissue is easier and biologically more robust compared to L1-promoter methylation assays. A 60% methylation is often used as a cut-off between promoter methylation and demethylation [30,31]. However, (i) some expressive tumors may have high methylation levels [38], as confirmed in our study; (ii) there is no clear-cut correlation between demethylation and transcription in tumor tissues [39]; and (iii) the deamination of cytosine into uracil, the central part of the sodium–bisulfite conversion protocol [40,41], represents one of the main artifacts of FFPE tissues [40], leading to potential errors.

L1-ORF expression using IHC on FFPE tissue is complex. A pioneering work about L1 expression in BC was based on IHC staining, allowing us to also evaluate the localization of the expression as a marker [17]; however, it was observed that not all L1 transcripts are translated into ORFs [41], and L1 sequences may play alternative roles as non-coding RNAs [42], leading to an underestimation of true L1 levels.

The application of an RNA-based test was not free from issues, mainly associated with the overlapping of L1 DNA–RNA sequences [43] and with the fragmentation of nucleic acids in FFPE tissues. Nevertheless, we applied a further DNase step to reduce the amount of residual DNA to a negligible level. To counteract the impact of fixation artifacts on the RNA and to simplify the L1 biomarker for statistical analysis, we considered as “L1 high-expressors” only patients with both ORFs over the median level of expression. This approach reduced the complexity, retaining the same value of clinical significance.

L1 reactivation in BC has historical proofs: in 1988 the insertion of L1 into the *MYC* gene showed the correlation between the hypomethylation of these sequences and a retrotransposon-linked carcinogenic effect [10]. Over the years, different L1 analysis approaches (methylation, RNA, alternative transcripts, protein expression and de novo insertions) confirmed the occurrence of L1 reactivation in BC [10,15,42,44].

Our results in the BC cell lines are in line with previous reports [16,45], thus confirming a statistically significant enrichment of L1 in basal-like cells. The association between L1 and BC aggressive features was firstly reported by Chen and colleagues, with a significant increase in protein expression in triple-negative breast cancers (TNBCs), associated with shorter OS [15]. This association between basal-like tumors and L1 overexpression was partially confirmed by McKerrow and colleagues [46], who reported an increased L1 expression in *TP53* mutated tumors, recently confirmed in breast, ovarian and colon cancers [46]. The present data further corroborate the correlation of L1 expression with highly proliferative and aggressive lesions [47].

Among all BC pathological factors, we highlighted a possible cross-link between L1 expression and immune-cell infiltration, since L1-high BCs showed high levels of TILs in the stroma. Histologic evaluation of TILs has reached level IB evidence as a prognostic marker in TNBC and level 2A with respect to prediction of response to chemotherapy. Some authors have shown a favorable outcome in TNBCs with TILs > 30% treated by surgery alone [48], thus suggesting a possible role in the de-escalation of treatment in this subset of patients. Nevertheless, at present, TIL levels are not used to withhold chemotherapy in BC patients. In addition, we should acknowledge that the current biomarker used in predicting responsiveness to immunotherapy in TNBC is PD-L1 [49,50], which is almost exclusively expressed in immune cells [51,52]. The role of L1 retrotransposon in the modulation of lymphocyte activation has been recently demonstrated by Marasca and colleagues [53], thus suggesting an implication of L1 expression in the context of cancer-immunology.

We also explored the possible clinical impact of L1 expression levels. High L1 levels significantly correlated with a higher risk of BC recurrence in terms of DFS. In the multivariate analysis, lymph-node involvement and L1 expression were the only two independent/significant variables. Node involvement is recognized as one of the main markers for the risk of relapse in BC [54,55,56]. By confirming the independence of L1 with respect to node involvement as a prognostic marker, retrotransposon expression could represent a high risk of relapse in BC patients.

By stratifying the risk according to the BC subtype, the relationship between HR-negative BCs and L1 expression showed stronger results, whereas HR-positive/L1-high lesions did not show a reduced DFS, suggesting poor biological and prognostic significance of L1 retrotransposons in Luminal tumors. HER2-enriched BCs showed great heterogeneity for L1 mRNA level, both in cell models and in the retrospective cohort. HER2-addicted tumors were also characterized by a significant, albeit less robust, L1 stratification of the risk of relapse.

Interestingly, L1 has also been considered from a therapeutic perspective. The use of reverse-transcriptase inhibitors (efavirenz) has been shown as particularly effective in TNBC preclinical models and is associated with the metabolism of fatty acids [45]. Conversely, resistance to paclitaxel in TNBC cell lines mediated by paclitaxel-induced L1 RNA stabilization was reversed by the use of efavirenz [57]. In our cohort, patients treated with chemotherapy and experiencing tumor relapse showed significantly higher L1 expression levels, and most of these were basal-like BCs.

Our work presents two main issues, both associated with the retrospective design. First, given the heterogeneity of the population, it is impossible to recover data about the overall survival. Second, the small amount of archival material precluded high-throughput DNA analysis for all tumors in the study cohort.

## 5. Conclusions

L1 expression could be interpreted as a strong negative prognostic marker, especially for HR-negative BCs. When considering the impact on treatment, our data may lead to a hypothesis generating further studies. Indeed, whether L1 expression may be exploited to modulate the treatment of TNBCs or to predict response to chemotherapy or to the combination of chemotherapy and ICIs warrants further investigation.

## Figures and Tables

**Table 1:** BC cohort composition and relative ORF1 and ORF2 expression comparisons.

		# of Patients (%)	ORF1_(RQ)_ Mean (SD)	*p*-Value	ORF2_(RQ)_ Mean ± SD	*p*-Value
PAM50 molecular subtype (*n* = 210)	Luminal A	67 (32%)	8.33 (7.6)	***p* < 0.0001**	12.99 ± 12.1	***p* < 0.0001**
	Luminal B	51 (24%)	12.93 (12.0)		19.13 ± 18.2	
	HER2-E	52 (25%)	21.86 (18.3)		31.12 ± 28.3	
	Basal-like	40 (19%)	34.04 (23.5)		58.76 ± 40.2	
Histotype (*n* = 210)	Ductal	16 (8%)	19.01 (16.6)	*p* = 0.34	28.03 (26.2)	*p* = 0.07
	Lobular	171 (81%)	8.30 (5.3)		12.08 (10.4)	
	Other	23 (11%)	14.40 (12.8)		23.62 (21.8)	
Ki67 (*n* = 210)	Ki67 < 20%	48 (23%)	10.30 (8.9)	***p* = 0.0024**	13.38 (11.9)	***p* = 0.0005**
	ki67 > 20%	162 (77%)	19.91 (14.2)		31.93 (25.3)	
Tumor size (*n* = 210)	<20 mm	71 (34%)	14.79 (11.4)	*p* = 0.11	24.08 (21.8)	*p* = 0.23
	>20 mm	139 (66%)	19.21 (16.8)		29.69 (22.9)	
Grade (*n* = 210)	G1–G2	69 (33%)	11.21 (9.9)	***p* = 0.0006**	17.80 (16.4)	***p* = 0.0019**
	G3	141 (67%)	20.87 (17.0)		32.53 (26.3)	
TILs (*n* = 210)	Low (0–10%)	127 (60%)	15.23 (14.80)	***p* = 0.02**	23.78 (17.1)	***p* = 0.01**
	Intermediate (10–30%)	58 (28%)	20.73 (18.2)		31.41 (24.9)	
	High (>30%)	25 (12%)	30.28 (26.5)		51.29 (46.95)	
Node (*n* = 210)	Negative	70 (33%)	14.24 (13.8)	*p* = 0.15	23.15 (21.2)	*p* = 0.38
	1–3 nodes	90 (43%)	18.51 (17.3)		30.43 (29.3)	
	>3 nodes	50 (24%)	21.00 (17.5)		29.97 (27.4)	
Relapse (*n* = 208)	Yes	112 (54%)	21.96 (20.7)	***p* = 0.0034**	34.02 (30.9)	***p* = 0.0096**
	No	96(46%)	14.13 (13.0)		22.33 (19.6)	

**Table 2:** Univariate and multivariate DFS results for L1 expression and clinicopathological features. Multivariate for non-L1 parameters refers to L1(RQ) median-containing Cox regression model.

		DFS				
			Univariate Analysis		Multivariate Analysis	
		Median Months (95% CI)	HR (95% CI)	*p*-Value	HR (95% CI)	*p*-Value
Grade	G1–G2	54 (1.4 to 128.4)	1		1	
	G3	44 (2.5 to 120.9)	1.7 (1.1 to 2.6)	**0.0089**	1.3 (0.8 to 2.2)	0.301
Histotype	Ductal	44 (1.4 to 121.1)	1		1	
	Lobular	47 (2.1 to 118.2)	1.0 (0.5 to 2.0)	0.983	1.2 (0.5 to 2.5)	0.746
	Other	41 (8.8 to 128.4)	1.0 (0.5 to 2.0)	0.934	1.1 (0.5 to 2.4)	0.634
Ki67	Ki67 < 20%	85 (1.4 to 118.2)	1		1	
	ki67 > 20%	42 (2.1 to 128.4)	1.3 (0.8 to 2.1)	0.260	1.1 (0.6 to 2.1)	0.501
Node	Negative	48 (7.7 to 128.4)	1		1	
	1–3 nodes	52 (1.4 to 119.7)	1.0 (0.5 to 1.6)	0.944	0.9 (0.5 to 1.5)	0.702
	>3 nodes	29 (5.0 to 118.2)	2.9 (1.7 to 4.9)	**0.0001**	2.3 (1.4 to 3.9)	**0.002**
PAM50 molecular subtype	Luminal A	51 (1.4 to 121.1)	1		1	
	Luminal B	49 (2.1 to 128.4)	1.0 (0.6 to 1.8)	0.996	0.8 (0.4 to 1.6)	0.528
	HER2-E	37 (5.0 to 119.7)	1.5 (0.9 to 2.7)	0.101	1.1 (0.6 to 2.3)	0.710
	Basal-like	34 (7.6 to 113.6)	2.0 (1.1 to 3.7)	**0.027**	1.3 (0.6 to 2.7)	0.568
TILs	Low (0–10%)	41 (1.4 to 128.4)	1		1	
	Intermediate (10–30%)	53 (6.0 to 120.9)	1.0 (0.6 to 1.7)	0.907	1.1 (0.6 to 1.7)	0.820
	High (>30%)	49 (5.0 to 111.9)	1.3 (0.6 to 2.6)	0.491	0.9 (0.4 to 1.8)	0.840
Tumor Size	<20 mm	54 (1.4 to 121.1)	1		1	
	>20 mm	38 (2.1 to 128.4)	1.7 (1.1 to 2.5)	**0.020**	1.5 (0.9 to 2.5)	0.067
ORF1_(RQ)_ Mean	Low	49 (1.4 to 128.4)	1		1	
	High	31 (2.1 to 121.2)	2 (1.3 to 3.1)	**0.0017**	1.7 (1.1 to 2.56)	**0.012**
ORF1_(RQ)_ Median	Low	49 (1.4 to 128.4)	1		1	
	High	37 (2.1 to 121.1)	1.7 (1.1 to 2.5)	**0.0172**	1.2 (1.1 to 1.5)	**0.043**
ORF2_(RQ)_ Mean	Low	49 (1.4 to 128.4)	1		1	
	High	36 (2.5 to 121.1)	1.9 (1.2 to 2.9)	**0.0065**	1.2 (1.1 to 1.5)	**0.037**
ORF2_(RQ)_ Median	Low	51 (1.4 to 128.4)	1		1	
	High	36 (2.1 to 121.1)	1.8 (1.4 to 2.5)	**0.0097**	1.3 (1.1 to 1.6)	**0.020**
L1_(RQ)_ Median	Low	50 (1.4 to 128.4)	1		1	
	High	35 (2.1 to 121.1)	2 (1.3 to 3.0)	**0.0014**	1.8 (1.2 to 2.7)	**0.005**
L1_(RQ)_ Mean	Low	48 (1.4 to 128.4)	1		1	
	High	36 (2.5 to 121.1)	1.8 (1.1 to 2.9)	**0.0089**	1.4 (1.1 to 1.7)	**0.038**

**Table 3:** Univariate DFS results for PAM50 molecular subtype in “L1 high-expressors”.

	DFS		
		Univariate analysis	
**PAM50 molecular subtype**	Median months (95% CI)	HR (95% CI)	*p*-value
Luminal A	54 (11.9 to 121.1)	1	
Luminal B	39 (2.1 to 120.9)	1.7 (0.5 to 5.4)	0.363
HER2-E	28 (5.0 to 116.9)	2.6 (1.1 to 6.1)	**0.027**
Basal-like	28 (7.6 to 111.7)	2.9 (1.3 to 6.4)	**0.0096**

## Data Availability

All data are included in the main manuscript and in the Supplementary Materials.

## Supplementary Materials

The following supporting information can be downloaded at: https://www.mdpi.com/article/10.3390/cells11121944/s1, Figure S1: (A) Correlation plot for ORF1 and methylation levels in the 41 BC tumors. (B) Correlation plot for ORF2 and methylation levels in the 41 BC tumors. (C) Boxplot reporting the expression of both ORF1 and ORF2 in PIK3CA/AKT mutated, WT or TP53 mutated BCs (*n* = 41). Figure S2: (A) Heatmap of ORF1(RQ) and ORF2(RQ) within BC, with recurrence (N = light blue, Y = dark blue) annotation, clustered for the median level (above or below). Green-to-red scale for the log2 expression revealed a sensible increased expression in patients with tumor relapse. (B) Patient relapse ROC curves for both ORF1 and ORF. Plots also reporting the area under the curve (AUC) with range.); Table S1: BC cell lines analyzed in the study, Table S2: BC cohort features; Table S3: Primers, thermal profiles and protocols for L1 analysis, Table S4: Mutation identified in the 41 BC patients; Table S5: L1 expression stratified by BC treatment (*n* = 207).
