# Supplementary material for: The Tumor-Specific Expression of L1 Retrotransposons Independently Correlates with Time to Relapse in Hormone-Negative Breast Cancer Patients
Source: Cells. 2022 Jun 16;11(12):1944. doi: 10.3390/cells11121944 (PMC9221920; doi:10.3390/cells11121944)
Supplement: Supplementary file 1 [file cells-11-01944-s001.zip › cells-1744865-supplementary.pdf]

cells

Supplementary Materials

The Tumor-Specific Expression of L1 Retrotransposons Independently Correlates with Time to Relapse in Hormone-Negative Breast Cancer Patients

Enrico Berrino [1,2,†], Umberto Miglio [1,†], Sara Erika Bellomo [1,3], Carla Debernardi [2], Alberto Bragoni [1,2], Annalisa Petrelli [1], Eliano Cascardi [1,2], Silvia Giordano [1,3], Filippo Montemurro [1], Caterina Marchiò [1,2], Tiziana Venesio [1,*] and Anna Sapino [1,2]

1 Candiolo Cancer Institute, FPO-IRCCS, 10060 Candiolo, Italy; enrico.berrino@ircc.it (E.B.); umberto.miglio@ircc.it (U.M.); saraerika.bellomo@unito.it (S.E.B.); alberto.bragoni@ircc.it (A.B.); annalisa.petrelli@unito.it (A.P.); eliano.cascardi@ircc.it (E.C.); silvia.giordano@unito.it (S.G.); filippo.montemurro@ircc.it (F.M.); caterina.marchio@unito.it (C.M.); anna.sapino@unito.it (A.S.)
2 Department of Medical Sciences, University of Turin, 10124 Turin, Italy; carla.debernardi@unito.it
3 Department of Oncology, University of Turin, 10124 Turin, Italy
* Correspondence: tiziana.venesio@ircc.it; Tel.: +39-011-9933547; Fax: +39-011-9933480
† These authors contributed equally to this work.

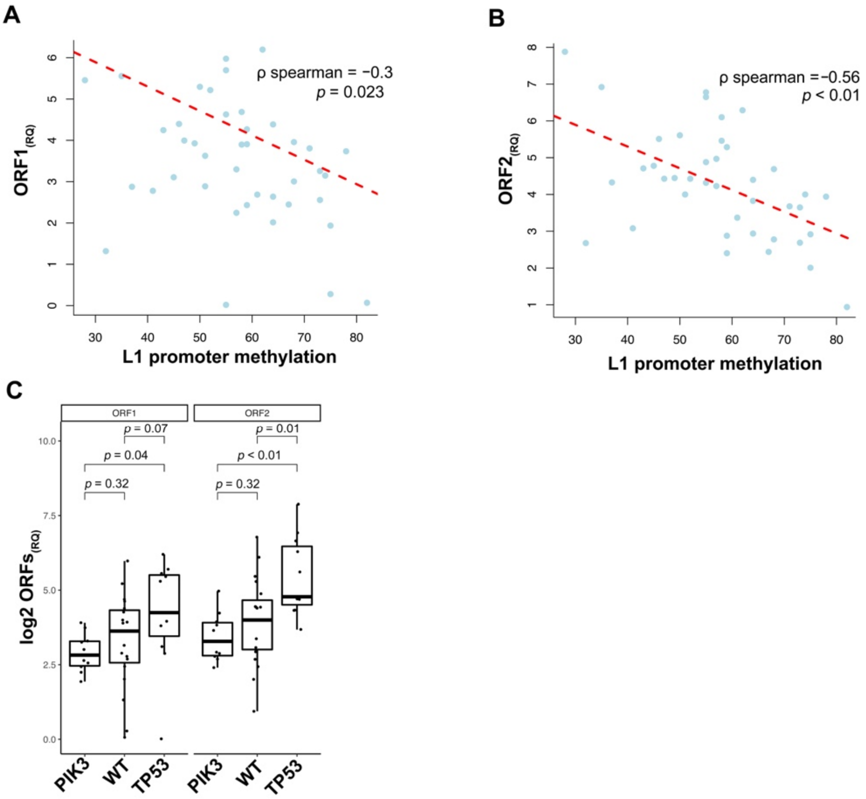

Figure S1. (**A**) Correlation plot for ORF1 and methylation levels in the 41 BC tumors. (**B**) Correlation plot for ORF2 and methylation levels in the 41 BC tumors. (**C**) Boxplot reporting the expression of both ORF1 and ORF2 in *PIK3CA*/*AKT* mutated, WT or *TP53* mutated BCs ($n = 41$).

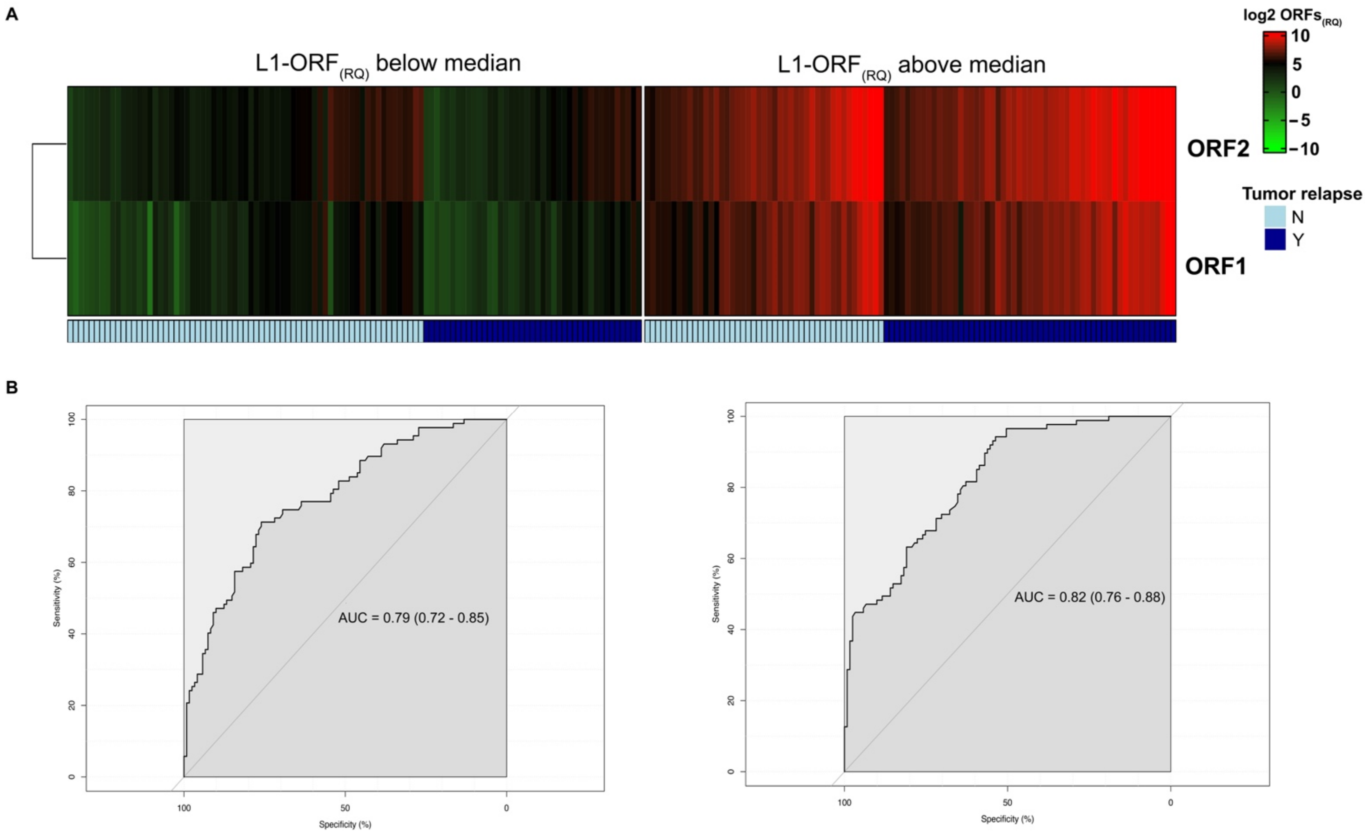

Figure S2. (A) Heatmap of ORF1(RQ) and ORF2(RQ) within BC, with recurrence (N = light blue, Y = dark blue) annotation, clustered for the median level (above or below). Green-to-red scale for the log2 expression revealed a sensible increased expression in patients with tumor relapse. (**B**) Patient relapse ROC curves for both ORF1 and ORF. Plots also reporting the area under the curve (AUC) with range.

Table S1. BC cell lines analyzed in the study.

Cell Line	PAM50	Histology	ER	PR	HER2	Metastatic Potential	ORF1 (dCT)	ORF2(dCT)	L1(dCT)
BT474	Luminal B	Invasive Ductal Carcinoma	+	+	+	1	0.29	0.09	0.19
BT549	Basal-like	Adenocarcinoma	-	-	-	3	0.55	0.39	0.47
HCC1187	Basal-like	Invasive Ductal Carcinoma	-	-	-	2	2.54	1.83	2.185
HCC70	Basal-like	Ductal Carcinoma	-	-	-	2	0.44	0.31	0.375
HMEC	Non transformed	Human Mammary	na	na	na	0	0.05	0.03	0.04

		Epithelial Cell								
HS578T	Basal-like	Invasive Ductal Carcinoma	-	-	-	3	1.42	0.88	1.15
MCF-7	Luminal A	Invasive Ductal Carcinoma	+	+	-	1	0.54	0.41	0.475
MCF10A	Non transformed	Fibrocystic disease	na	na	na	0	0.09	0.14	0.115
MDA-MB 231	Basal-like	Adenocarcinoma	-	-	-	1	0.59	0.31	0.45
MDA-MB 453	HER2	Adenocarcinoma	-	-	+	3	1.04	0.91	0.975
MDA-MB-468	Basal-like	Ductal Carcinoma	-	-	-	1	1.62	0.89	1.255
SKBR3	HER2	Adenocarcinoma	-	-	+	1	0.25	0.11	0.18
T47D	Luminal A	Invasive Ductal Carcinoma	+	+	-	1	0.47	0.4	0.435
ZR751	Luminal A	Invasive Ductal Carcinoma	+	+/-	-	1	0.09	0.03	0.06

Table S2. BC cohort features.

Age (*n* = 210)	Mean
	58 (range 30-89)
Ki67 (*n* = 210)	Mean (%)
	38.5 (range 3-95)
TILs (*n* = 210)	Mean (%)
	10 (range 2-60)
Tumor Size (*n* = 210)	Mean (mm)
	26.5 (range 2-90)
Histotype (*n* = 210)	# Patients (%)
Lobular	16 (7.5%)
Ductal	171 (81.5%)
Other	23 (11%)
Grading (*n* = 210)	
G1	5 (2.5%)
G2	64 (30.5%)
G3	141 (67%)
Staging (*n* = 210)	
pT1	69 (33%)
pT2	116 (55%)
pT3	17 (8%)
pT4	8 (4%)
Lymph node metastases (*n* = 210)	
pN0	69 (33%)
pN1	114 (54%)
pN2	14 (6.5)
pN3	13 (6%)
Molecular subtype (*n* = 210)	
Luminal A	67 (32%)
Luminal B	51 (24%)
HER2	52 (25%)
Basal-like	40 (19%)
Recurrence (*n* = 210)	
Yes	112 (53%)
No	96 (46%)
Unknown	2 (1%)
Treatment (*n* = 209)	
Chemotherapy	160 (76%)
Radiotherapy	157 (75%)
Hormone therapy	156 (74%)
Anti-HER2 therapy	48 (23%)
Relapse (*n*= 208)	
Yes	96 8 (46%)
No	112 (54%)

Table S3. Primers, thermal profiles and protocols for L1 analysis.

	**qRT-PCR and Methylation Primers**
	5'-GGC CAA CGT TCA GAT TCA GG-3'
ORF1-R	5'-AAC CCG ACC TTT CTC TCT GG-3'
ORF1-RA	5'-AAC CCG ACC TTT CTC TCT GG-3'
ORFA-FA	5'-GGC CAA CGT TCA GAT TCA GG-3'
GAPDH-F	5'-GAA GGT GAA GGT CGG AGT C-3'
GAPDH-R	5'-GAA GAT GGT GAT GGG ATT TC-3'
18S-F	5'-AGTCCCTGCCCTTTGTACACA-3'

18S-R	5'-GATCCGAGGGCCTCACTAAAC-3'		
L1 methylation-F	5′-GAGTTAGGTGTGGGATATAGT-3′		
L1 methylation-R	5′biotin-CAAAAAATCAAAAAA TTCCCTTTCC-3′		
L1 methylation sequencing	5′-GGTGTGGGATATAGTT-3′.		
L1-ORF1	Concentration		95°C (3 minutes)
cDNA	50 ng		95°C (3 minutes)
H20	to 25 ul	30 cycles	95°C (30 seconds)
MgCl2	3mM		63°C (30 seconds)
dNTPs	0,2mM		72°C (45 seconds)
Buffer	1x		72°C (5 minutes)
PF	0,4 uM		
PR	0,4 uM		
TakaraTaq	1U		
EvaGreen	2X		
L1-ORF2	Concentration		95°C (3 minutes)
cDNA	50 ng		95°C (3 minutes)
H20	to 25 ul	30 cycles	95°C (30 seconds)
MgCl2	3mM		60°C (30 seconds)
dNTPs	0,2mM		72°C (45 seconds)
Buffer	1x		72°C (5 minutes)
PF	0,4 uM		
PR	0,4 uM		
TakaraTaq	1U		
EvaGreen	2X		
18S	Concentration		95°C (3 minutes)
cDNA	50 ng		95°C (3 minutes)
H20	to 25 ul	25 cycles	95°C (30 seconds)
MgCl2	2mM		58°C (30 seconds)
dNTPs	0,2mM		72°C (45 seconds)
Buffer	1x		72°C (5 minutes)
PF	0,4 uM		
PR	0,4 uM		
TakaraTaq	1U		
EvaGreen	2X		
GAPDH	Concentration		95°C (3 minutes)
cDNA	50 ng		95°C (3 minutes)
H20	to 25 ul	30 cycles	95°C (30 seconds)
MgCl2	2mM		58°C (30 seconds)
dNTPs	0,2mM		72°C (45 seconds)
Buffer	1x		72°C (5 minutes)
PF	0,4 uM		
PR	0,4 uM		
TakaraTaq	1U		
EvaGreen	2X		
**L1-promoter methylation**			

	Concentration		
Coverted DNA	50 ng		95°C (3 minutes)
H20	to 25 ul		95°C (25 seconds)
MgCl2	3mM	40 cycles	50°C (25 seconds)
dNTPs	0.2 mM		72°C (25 seconds)
Buffer	1x		72°C (5 minutes)
PF	0,4 uM		
PR	0,4 uM		
GoTaq	1.25U		
EvaGreen	2X		

Table S4. Mutation identified in the 41 BC patients.

Patient ID	Mutated Gene	Coding Mutation	Protein Mutation
1	*PIK3CA*	c.1035T>A	p.N345K
2	*TP53*	c.574C>T/743G>A	p.Q192*/p.R248Q
3	*WT*	/	/
4	*PIK3CA*	c.3140A>G	p.H1047R
5	*PIK3CA*	c.1637A>C	p.Q546P
6	*PIK3CA*	c.3140A>T	p.H1047L
7	*AKT*	c.49G>A	p.E17K
8	*PIK3CA*	c.1634A>G	p.E545G
9	*PIK3CA*	c.3140A>T	p.H1047L
10	*TP53*	c.818G>A	p.R273H
11	*WT*	/	/
12	*TP53*	c.586C>T	p.R196Ter
13	*WT*	/	/
14	*AKT*	c.49G>A	p.E17K
15	*WT*	/	/
16	*WT*	/	/
17	*WT*	/	/
18	*TP53*	c.578A>G	p.H193r
19	*PIK3CA*	c.1634A>G	p.E545G
20	*WT*	/	/
21	*PIK3CA*	c.3140A>G	p.H1047R
22	*WT*		/
23	*TP53*	c.711G>A	p.M237I
24	*WT*	/	/
25	*WT*	/	/
26	*WT*	/	/
27	*WT*	/	/
28	*WT*	/	/
29	*CDH1*	c.1774G>A	p.A542T
30	*WT*	/	/
31	*WT*	/	/
32	*MAP2K4*	c.946C>T	p.Q316*
33	*TP53*	c.637C>T	p.R213Ter
34	*TP53*	c.586C>T	p.R196Ter
35	*TP53*	c.584T>C	p.I195T
36	*WT*	/	/
37	*TP53*	c.742C>T	p.R248W
38	*WT*	/	/
39	*TP53*	c.742C>T/c.574C>T	p.Q192Terp.R248W

40	*TP53*	c.637C>T	p.R213Ter
41	*TP53*	c.637C>T	p.R213Ter

Table S5. L1 expression stratified by BC treatment (n = 207).

		Relapse	No Relapse	
Chemotherapy alone (n = 50)		n=29	n=21	p-value
	ORF1(RQ)	35.18	22.23	**0.03**
	ORF2(RQ)	57.32	35.55	**0.03**
Chemo + Hormone therapy (n = 80)		n= 38	n= 52	p-value
	ORF1(RQ)	16.84	11.58	0.9
	ORF2(RQ)	23.21	16.18	0.9
Hormone therapy alone (n = 31)		n=13	n=16	p-value
	ORF1(RQ)	7.9	10.74	0.4
	ORF2(RQ)	13.86	17.74	0.9
Anti-HER2 therapy (n = 46)		n= 25	n= 21	p-value
	ORF1(RQ)	23.71	11.19	0.2
	ORF2(RQ)	33.59	28.19	0.5
